# Does the Integration of Inflammatory Markers with Clinical Scoring Systems Improve the Prediction of Neurosurgical Intervention in Emergency Department Patients Diagnosed with Traumatic Subarachnoid Hemorrhage?

**DOI:** 10.3390/diagnostics16182949

**Published:** 2026-09-12

**Authors:** Sema Ayten, Vehbi Özaydın, Ayça Yılmaz Atinkaya, Emine Ünal

**Affiliations:** 1 Department of Emergency Medicine, Istanbul Göztepe Prof. Dr. Süleyman Yalçın City Hospital, 34722 Istanbul, Türkiye; vozaydin@hotmail.com (V.Ö.); draycayilmaz@gmail.com (A.Y.A.); 2 Department of Emergency Medicine, Istanbul Bahçelievler State Hospital, 34180 Istanbul, Türkiye; unalemine94@gmail.com

**Keywords:** traumatic subarachnoid hemorrhage, neurosurgical procedures, inflammatory markers, white blood cell count, Glasgow Coma Scale, Injury Severity Score, emergency department, receiver operating characteristic curve

## Abstract

**Background/Objectives:** Traumatic subarachnoid hemorrhage (tSAH) is one of the most frequent intracranial findings in moderate-to-severe traumatic brain injury. This study evaluated whether integrating systemic inflammatory markers—white blood cell count (WBC), neutrophil count, neutrophil-to-lymphocyte ratio (NLR), and C-reactive protein (CRP)—with the Glasgow Coma Scale (GCS) and Injury Severity Score (ISS) improves the prediction of neurosurgical intervention in adult tSAH patients, compared with clinical scores alone. **Methods:** This single-center retrospective cohort study included 69 adult patients (age ≥ 18 years) with computed tomography (CT)-confirmed tSAH treated between January and December 2023. Patients younger than 18 years were excluded a priori given age-specific reference ranges for WBC and lymphocyte count. Neurosurgical intervention was defined as any cranial or therapeutic intracranial vascular procedure directly addressing the intracranial pathology; patients whose only procedure was unrelated to the intracranial injury were classified as non-surgical. Patients were stratified into surgery (*n* = 12) and no-surgery (*n* = 57) groups. Diagnostic accuracy was assessed by receiver operating characteristic (ROC) curve analysis with DeLong pairwise comparisons; logistic regression estimated odds ratios (ORs). **Results:** WBC (area under the curve [AUC] = 0.850), GCS (AUC = 0.831), and ISS (AUC = 0.827) were the strongest individual predictors; neutrophil count was also significant (AUC = 0.807), while NLR and CRP were not discriminatory. The combined WBC + GCS model achieved an AUC = 0.904 (sensitivity 75.0%, specificity 93.0%), without statistically significant superiority over either predictor alone (DeLong *p* = 0.571 and *p* = 0.455, respectively). In multivariable analysis, both WBC (OR = 3.951, *p* = 0.006) and GCS (OR = 0.331, *p* = 0.002) were independent predictors of neurosurgical intervention. A sensitivity analysis excluding the single endovascular-intervention case (*n* = 68) yielded materially unchanged results. **Conclusions:** WBC and GCS were independently associated with neurosurgical intervention in this exploratory adult tSAH cohort. The combined model showed numerically higher discrimination than either predictor alone, although this incremental difference was not statistically significant. Given the small event count, these findings should be regarded as hypothesis-generating; internal and external validation in larger cohorts is required before clinical application.

## 1. Introduction

Traumatic brain injury (TBI) represents a major global public health burden, accounting for a substantial proportion of emergency department (ED) visits and being associated with high rates of mortality and long-term disability, particularly among young adults [1]. Traumatic subarachnoid hemorrhage (tSAH)—defined as the presence of blood in the subarachnoid space resulting from external mechanical force—is the most common intracranial hemorrhagic complication of TBI, detected in 33–60% of moderate-to-severe TBI cases on cranial computed tomography (CT) [2]. Although the majority of tSAH cases are managed conservatively, a clinically significant subset requires neurosurgical intervention, typically for complications such as hydrocephalus [3] or an associated vascular lesion [2].

Accurate and timely identification of which tSAH patients will require neurosurgical management remains an important focus of emergency care, particularly as most tSAH cases follow a benign course and only a minority ultimately require neurosurgical intervention [2]. Traditional clinical assessment tools—the Glasgow Coma Scale (GCS) for rapid neurological evaluation and the Injury Severity Score (ISS) for quantifying polytrauma burden—have formed the cornerstone of triage decision-making in trauma; comparative validation of these scores against mortality and functional outcomes, however, has been reported primarily in pediatric cohorts, whereas the present study concerns adult patients [4,5]. Nevertheless, accurate GCS assessment may not be feasible in certain clinical circumstances—namely, when patients with severe head injury require intubation for airway protection, pharmacological paralysis for intracranial pressure management, or sedation to ensure compliance during cranial CT acquisition in agitated patients [6,7]. In addition, these instruments primarily reflect the direct structural consequences of injury, whereas TBI is also known to trigger a systemic inflammatory response that is not captured by clinical severity scores alone [8]. Furthermore, while ISS is a well-validated severity measure, its calculation requires complete injury documentation and is therefore not immediately available at the point of initial triage [9].

Trauma induces a systemic inflammatory response syndrome characterized by leukocytosis [8]; admission white blood cell count (WBC) has been reported as associated with the presence of significant intracranial lesions in trauma [10]. NLR has been investigated as a prognostic biomarker, particularly in traumatic brain injury [11,12]. Under normal conditions, the blood–brain barrier restricts leukocyte entry into the brain; disruption of this barrier following TBI permits early neutrophil infiltration into the central nervous system through the choroid plexus and meningeal vasculature, occurring in parallel with a marked rise in peripheral neutrophil counts within the first 48 h after injury [11]. NLR, which integrates neutrophilia and stress-related lymphopenia, has been specifically studied as a prognostic marker in TBI [11,12,13].

A growing body of evidence suggests that post-traumatic neutrophilia and elevated NLR are associated with unfavorable functional outcomes following TBI [12,13], although evidence for an independent association between NLR and mortality specifically remains inconsistent: a retrospective cohort with updated meta-analysis in aneurysmal subarachnoid hemorrhage found NLR to be independently associated with poor outcome [14], whereas at least one other meta-analysis found no significant NLR–mortality association despite substantial heterogeneity [13]. Despite this biological rationale, studies evaluating the combined predictive value of inflammatory markers and clinical scores specifically for the endpoint of neurosurgical intervention in tSAH remain scarce. The present study was designed to investigate whether such integration provides higher diagnostic accuracy for identifying tSAH patients requiring neurosurgical intervention than clinical scores alone. We hypothesized that a combined model incorporating WBC with GCS—both available at initial ED assessment—would outperform either parameter individually.

## 2. Materials and Methods

### 2.1. Study Design and Setting

This single-center retrospective cohort study was conducted at Istanbul Göztepe Prof. Dr. Süleyman Yalçın City Hospital, a tertiary academic teaching hospital with a high-volume urban ED. The study period encompassed all tSAH cases presenting between 1 January and 31 December 2023. The study was approved by the local Institutional Ethics Committee (Approval No. 2026/0113) and conducted in accordance with the Declaration of Helsinki. Given the retrospective design and use of de-identified data, individual informed consent was waived by the ethics committee. This study was reported in accordance with the Strengthening the Reporting of Observational Studies in Epidemiology (STROBE) guidelines for observational research [15]. As this study also involved development of a multivariable prediction model, it was additionally guided by the Transparent Reporting of a multivariable prediction model for Individual Prognosis or Diagnosis (TRIPOD) statement [16]; per the TRIPOD classification, this corresponds to a Type 1b study (model development with resampling-based internal validation, but no external validation in a separate dataset).

### 2.2. Study Population

Eligible patients were identified through a systematic search of the hospital information system using International Classification of Diseases, 10th Revision (ICD-10) codes and radiological referral diagnoses. Adult patients (age ≥ 18 years) were included if they presented to the ED following trauma, had CT-confirmed tSAH at the index visit, and had a complete blood count and biochemical panel available at admission. Patients younger than 18 years of age were excluded a priori, as pediatric reference ranges for WBC and lymphocyte count differ substantially from adult values. Of 1884 patients with neurosurgical consultation requested in the ED during the study period, 148 had CT-confirmed tSAH; 71 of these were excluded for incomplete medical records, active infection/autoimmune disease, or corticosteroid/immunosuppressant use, and a further 8 pediatric patients were excluded, yielding the final analytic cohort of 69 adult patients. A patient-selection flow diagram is provided in Supplementary Figure S1.

### 2.3. Inclusion and Exclusion Criteria, and Outcome Definition

Neurosurgical intervention, rather than mortality, functional outcome, or radiological progression, was selected as the primary outcome for three reasons. First, it represents the most immediate, actionable decision facing emergency physicians at the point of initial assessment—whether a patient requires urgent neurosurgical referral—rather than a distal outcome influenced by numerous downstream factors (e.g., comorbidities, complications, rehabilitation access) only partially related to the index tSAH. Second, in this retrospective single-center cohort, mortality was a rare event, which would have provided even less statistical power than the 12 neurosurgical-intervention events analyzed here, precluding meaningful multivariable analysis. Third, radiological progression and standardized functional outcome scores (e.g., Glasgow Outcome Scale) were not systematically documented in the medical records available for this retrospective review, precluding their use as outcomes without introducing substantial missing-data bias.

Patients were excluded if they had a documented history of active infection or inflammatory/autoimmune disease at presentation, were receiving corticosteroids or immunosuppressive agents prior to admission, or had incomplete medical records.

Neurosurgical intervention was defined as any cranial or therapeutic intracranial vascular procedure performed directly to address the intracranial pathology, including craniotomy, craniectomy, external ventricular drain (EVD) placement, hematoma evacuation, aneurysm clipping, or therapeutic (interventional) intracranial/cervicocranial vascular procedures. Diagnostic angiography without a concurrent therapeutic component (e.g., embolization, stenting, or angioplasty) would not, by itself, meet this definition. Patients whose only surgical procedure during the index hospitalization was unrelated to the intracranial injury (e.g., isolated extremity fracture fixation) were classified as not having undergone neurosurgical intervention for the primary outcome.

### 2.4. Data Collection

Electronic medical records were reviewed for: (i) demographic and clinical data—age, sex, and comorbidities (diabetes mellitus, hypertension, active malignancy); (ii) trauma data—mechanism of injury, GCS at ED presentation, and ISS; (iii) laboratory data at ED admission—absolute neutrophil count, absolute lymphocyte count, WBC (all in 10^3^/µL), and serum CRP (mg/L); NLR was calculated as the neutrophil-to-lymphocyte ratio; and (iv) outcome—neurosurgical intervention during the index hospitalization, as defined in Section 2.3 (1 = yes, 0 = no).

### 2.5. Statistical Analysis

Analyses were performed using SPSS version 25.0 (IBM Corp., Armonk, NY, USA). Normality was assessed with the Shapiro–Wilk test; as all variables deviated significantly from normality in at least one group, non-parametric methods were used throughout. Continuous variables are presented as median (interquartile range [IQR]) and compared with the Mann–Whitney U test. Categorical variables are presented as *n* (%) and compared with Fisher’s exact test; for the trauma-mechanism contingency table (five categories), the chi-square test and, given expected cell counts < 5, a Monte Carlo estimate of the Fisher–Freeman–Halton exact test were additionally applied. Diagnostic accuracy was assessed by ROC analysis; AUC values are reported with 95% confidence intervals (CIs) by the Hanley–McNeil method. Study-derived, exploratory cut-offs—maximizing the Youden index in this dataset, and not intended as externally validated clinical thresholds—are reported alongside each AUC. Pairwise AUC comparisons used the DeLong method.

Univariable and multivariable binary logistic regression were performed; ORs are expressed per 1 standard deviation (SD) with 95% CIs derived from the Wald test based on the Fisher information matrix. Candidate predictors for the primary multivariable model were first narrowed to those with the highest individual AUC and immediate availability at ED triage—WBC, GCS, and neutrophil count. These three candidates were then screened for collinearity: neutrophil count was excluded due to very high collinearity with WBC (r = 0.921), leaving WBC and GCS (r = −0.365) as the final primary model. Lymphocyte count showed only moderate collinearity with WBC (r = 0.407) but was not considered for the primary model given its lower individual AUC and the events-per-variable constraint. ISS was evaluated separately in a secondary model (WBC + ISS) due to its limited real-time availability; ISS was not combined with GCS due to high collinearity (r = −0.680). Given *n* = 12 neurosurgical events, the events-per-variable (EPV) ratio (EPV = 6.0) limited the primary model to two predictors. A post hoc power calculation based on the observed AUC values is reported for descriptive completeness; however, such calculations are derived from the same effect sizes being tested and therefore do not independently establish that the sample size was adequate [17]. A *p*-value < 0.05 was considered statistically significant.

### 2.6. Use of Generative Artificial Intelligence

A generative artificial intelligence tool (Claude Sonnet 5, Anthropic, PBC, San Francisco, CA, USA) was used during preparation of this study to assist with statistical computation and independent verification, literature search support and language editing of the manuscript text. Specifically, AI assistance was used to perform exploratory and confirmatory statistical computations (descriptive statistics, ROC/AUC analysis, DeLong pairwise comparisons, univariable and multivariable logistic regression, and bootstrap internal validation) from the source dataset provided by the authors; every statistical output was independently cross-checked by the authors against the raw dataset and, for key analyses, against manual or spreadsheet-based recalculation, before inclusion in the manuscript. AI assistance was also used to search literature, with every cited source subsequently retrieved and verified by the authors for accuracy of content and citation. All interpretive and clinical content was reviewed, edited, and approved by the authors, who take full responsibility for the accuracy and integrity of the content of this publication.

## 3. Results

### 3.1. Patient Characteristics

A total of 69 adult patients with CT-confirmed tSAH were included. Of these, 12 (17.4%) underwent neurosurgical intervention and 57 (82.6%) were managed conservatively. The overall cohort had a median age of 65.0 years (IQR 42.0–78.0) and comprised 45 male (65.2%) and 24 female (34.8%) patients, with no significant between-group difference in sex distribution (*p* = 0.521). The most common mechanism of injury was same-level fall (*n* = 39, 56.5%), followed by fall from height (*n* = 13, 18.8%), assault (*n* = 8, 11.6%), and pedestrian traffic accident (*n* = 7, 10.1%); the distribution of trauma mechanism did not differ significantly between groups (*p* = 0.265, chi-square; *p* = 0.241, Monte Carlo estimate of the Fisher–Freeman–Halton exact test, 50,000 simulations). Comorbidities included hypertension (49.3%), diabetes mellitus (24.6%), and active malignancy (1.4%). No significant between-group difference was observed for age in this cohort (55.0 vs. 68.0 years; *p* = 0.289). Baseline characteristics stratified by intervention status are presented in Table 1.

A post hoc power calculation based on the observed AUC values (98.7–100% for WBC, GCS, ISS, neutrophil count, and the combined model) is reported for descriptive completeness; however, such calculations do not independently establish sample-size adequacy. Given the small event count (*n* = 12), the wide confidence intervals accompanying all AUC and OR estimates provide a more informative indication of the precision achievable in this cohort than the post hoc power figures. To further characterize potential overfitting, internal validation of the primary WBC + GCS model was performed using Harrell’s bootstrap optimism-correction method (2000 resamples): the apparent AUC of 0.904 corresponded to an optimism-corrected AUC of 0.896 (mean optimism = 0.007; 95% percentile range for optimism, −0.101 to 0.090), suggesting that overfitting was modest despite the low events-per-variable ratio, although meaningful uncertainty in this correction itself remains given the small sample.

### 3.2. Between-Group Comparison

**Figure 1:**
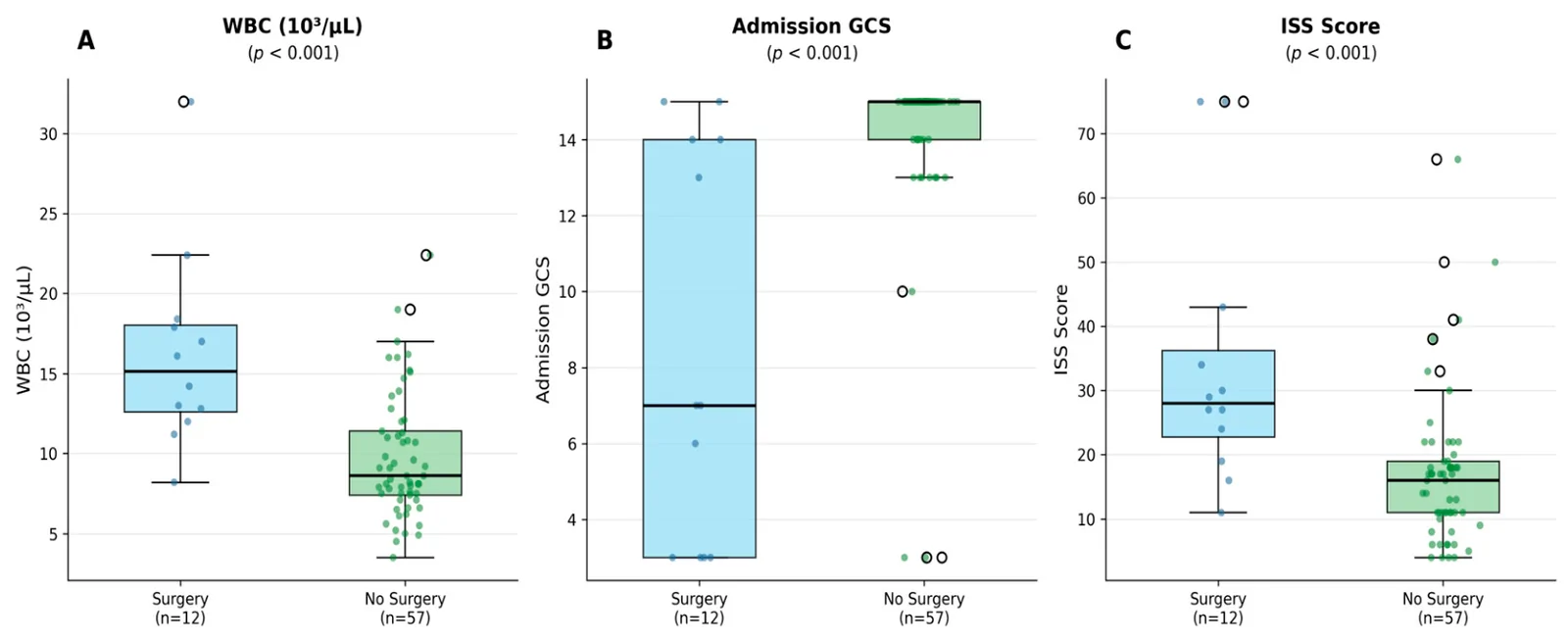
Box plots with individual data points comparing WBC, admission GCS, and ISS between neurosurgical intervention and no-intervention groups. *p*-values from the Mann–Whitney U test.

Patients who underwent neurosurgical intervention had significantly higher WBC (15.15 [12.60–18.02] vs. 8.60 [7.40–11.40] × 10^3^/µL; *p* < 0.001) and neutrophil counts (12.55 [7.36–16.13] vs. 5.67 [4.19–8.89] × 10^3^/µL; *p* = 0.001) than conservatively managed patients. Lymphocyte count was also significantly higher in the surgery group (2.55 [1.50–4.45] vs. 1.70 [1.30–2.50] × 10^3^/µL; *p* = 0.049). Admission GCS was significantly lower (7.0 [3.0–14.0] vs. 15.0 [14.0–15.0]; *p* < 0.001) and ISS higher (28.0 [22.8–36.3] vs. 16.0 [11.0–19.0]; *p* < 0.001) in the surgery group. No significant between-group differences were observed for NLR (*p* = 0.443) or CRP (*p* = 0.384). Findings are summarized in Table 1 and Figure 1.

### 3.3. Diagnostic Accuracy: ROC Analysis

**Figure 2:**
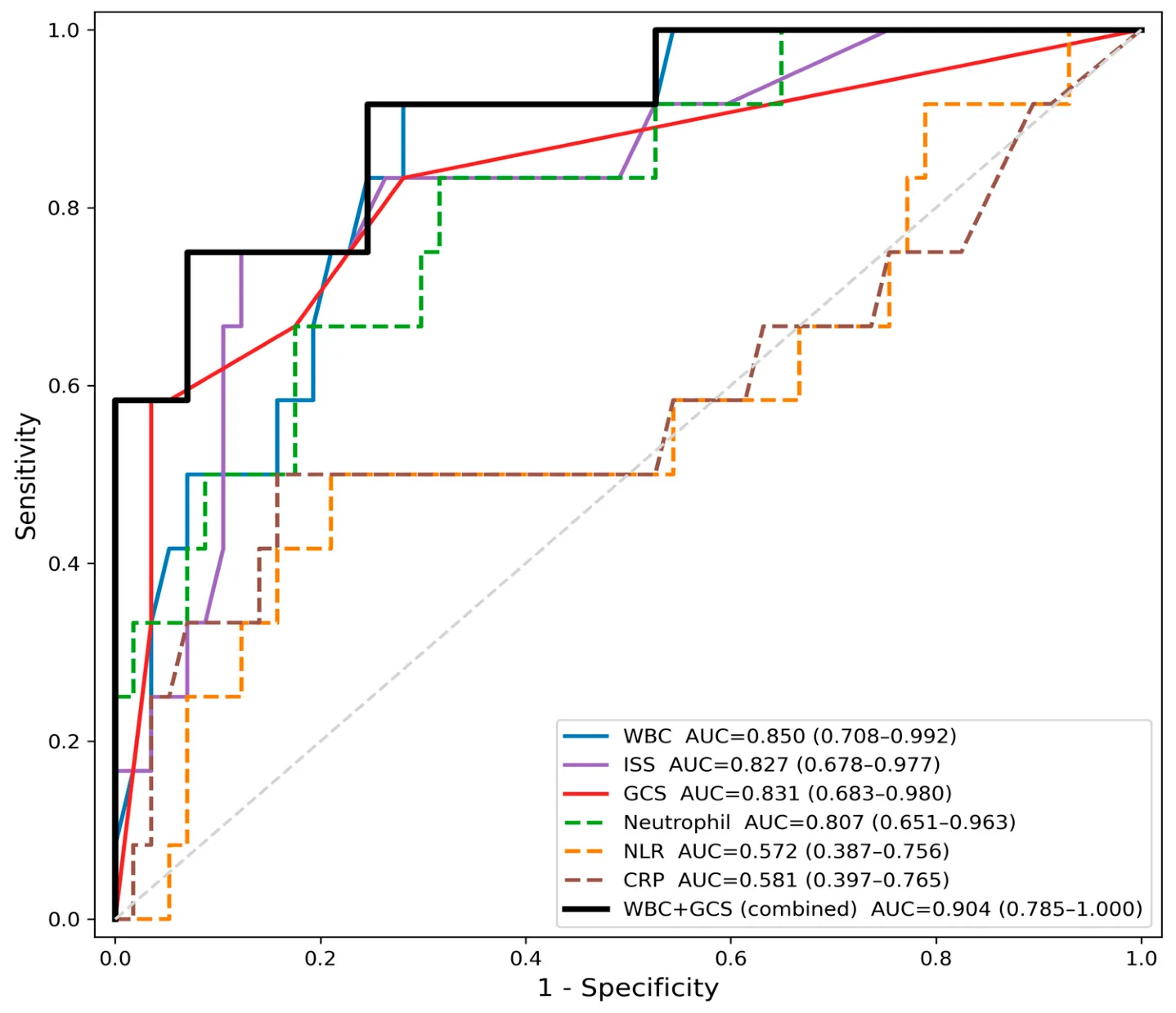
ROC curves for predicting neurosurgical intervention in tSAH (*n* = 69, surgery *n* = 12). AUC values with 95% CI (Hanley–McNeil). The primary combined WBC + GCS model (black line; AUC = 0.904) did not differ significantly from WBC alone (*p* = 0.571) or GCS alone (*p* = 0.455) by pairwise DeLong testing.

Table 2 summarizes the ROC analysis results. WBC achieved the highest individual AUC (0.850, 95% CI 0.708–0.992; cut-off > 11.20 × 10^3^/µL; sensitivity 91.7%, specificity 71.9%), followed by GCS (AUC = 0.831, 95% CI 0.683–0.980; cut-off ≤ 14; sensitivity 83.3%, specificity 71.9%), ISS (AUC = 0.827, 95% CI 0.678–0.977; cut-off > 24; sensitivity 75.0%, specificity 87.7%), and neutrophil count (AUC = 0.807, 95% CI 0.651–0.963). NLR (AUC = 0.572) and CRP (AUC = 0.581) did not achieve statistically significant discriminatory capacity. ROC curves are displayed in Figure 2.

The primary combined model (WBC + GCS) achieved AUC = 0.904 (95% CI 0.785–1.000; sensitivity 75.0%, specificity 93.0%)—the highest combined diagnostic performance observed across all analyses in this study. Pairwise DeLong testing showed a numerically superior but statistically non-significant improvement over either predictor alone (vs. WBC: Z = 0.566, *p* = 0.571; vs. GCS: Z = 0.747, *p* = 0.455); the study may have been insufficiently precise to exclude a clinically relevant incremental effect, but an incremental benefit was not statistically demonstrated and this numerical difference should not be over-interpreted. The secondary model (WBC + ISS; r = 0.235) achieved AUC = 0.886 (95% CI 0.759–1.000; sensitivity 100.0%, specificity 64.9%), but ISS is not immediately available at initial ED triage.

### 3.4. Logistic Regression Analysis

In univariable logistic regression (Table 3), WBC (OR = 3.907, 95% CI 1.696–9.000, *p* = 0.001), neutrophil count (OR = 3.216, 95% CI 1.599–6.469, *p* = 0.001), ISS (OR = 2.735, 95% CI 1.381–5.418, *p* = 0.004), GCS (OR = 0.302, 95% CI 0.160–0.567, *p* < 0.001), and lymphocyte count (OR = 1.787, 95% CI 1.023–3.125, *p* = 0.042) were statistically significant predictors. In the primary multivariable model, both WBC (OR = 3.951, 95% CI 1.473–10.597, *p* = 0.006) and GCS (OR = 0.331, 95% CI 0.167–0.656, *p* = 0.002) remained independently associated with neurosurgical intervention (combined AUC = 0.904). In the secondary model, both WBC (OR = 3.949, 95% CI 1.595–9.779, *p* = 0.003) and ISS (OR = 2.610, 95% CI 1.190–5.502, *p* = 0.012) were also independently significant. The complete fitted equation for the primary model, expressed in raw predictor units, was: Logit(P) = −1.3676 + (0.2764 × WBC) + (−0.3051 × GCS), where WBC is expressed in 10^3^/µL and GCS as the total score; predicted probability is obtained as P = 1/(1 + e^−x^), where x is the logit value. At the Youden-optimal probability threshold (0.27), the primary model correctly classified 62 of 69 patients (accuracy 89.9%), with a positive predictive value of 69.2% (95% CI 42.4–87.3%), negative predictive value of 94.6% (95% CI 85.4–98.2%), positive likelihood ratio of 10.69, and negative likelihood ratio of 0.27. Model calibration was also assessed: calibration-in-the-large showed close agreement between the mean predicted probability and the observed event rate (both 0.174). A 3-group Hosmer–Lemeshow test was not statistically significant (χ^2^ = 3.19, df = 1, *p* = 0.074), although this test is likely underpowered given the small event count and should be interpreted with caution. Decision curve analysis across threshold probabilities of 5–50% showed that the WBC + GCS model provided positive net benefit throughout this range and outperformed a treat-all strategy at threshold probabilities above approximately 15%, while consistently outperforming a treat-none strategy (net benefit = 0); as with all performance metrics reported here, this reflects apparent, in-sample performance in a small single-center cohort rather than externally validated clinical utility.

### 3.5. Types of Neurosurgical Intervention Performed and Sensitivity Analysis

Among the 12 patients who underwent neurosurgical intervention, 8 (66.7%) underwent an isolated cranial neurosurgical procedure (craniotomy, craniectomy, EVD placement, hematoma evacuation, or aneurysm clipping), 3 (25.0%) underwent a cranial neurosurgical procedure combined with an orthopedic procedure, and 1 (8.3%) underwent a therapeutic intracranial vascular intervention (carotid digital subtraction angiography with endovascular treatment) without craniotomy, craniectomy, or EVD placement. Chart review confirmed that this procedure was performed for a trauma-related vascular pathology directly associated with the tSAH, rather than for an incidental or unrelated finding, and therefore met the pre-specified definition of neurosurgical intervention. Although these 12 interventions were procedurally heterogeneous (isolated cranial procedures, cranial procedures combined with orthopedic surgery, and one endovascular procedure), all were performed emergently for acute, life-threatening mass effect or vascular pathology directly attributable to the tSAH, providing a shared clinical rationale for their inclusion under a single “neurosurgical intervention” outcome despite differing surgical techniques. To directly characterize this heterogeneity, the CT-documented indication underlying each intervention was reviewed (Table 4). Eleven of the 12 interventions (91.7%) were performed in the presence of an identifiable structural lesion accompanying the tSAH—most commonly an intraparenchymal or intraventricular hematoma, subdural hematoma, or significant mass effect (midline shift or diffuse edema)—rather than for tSAH volume alone; in no case was the surgical report or CT description framed as addressing the subarachnoid blood itself as the primary indication. One case (File 1622235) additionally demonstrated a cerebral aneurysm on angiography; because this raises the possibility of a co-existing or aneurysm-related hemorrhagic component rather than a purely traumatic mechanism, this case was reviewed in detail (see Discussion). This pattern is consistent with the broader tSAH literature, in which isolated tSAH—without an accompanying structural lesion—is only rarely the sole driver of neurosurgical intervention [18]; the present findings should therefore be interpreted as characterizing predictors of neurosurgical intervention in tSAH-inclusive TBI presentations broadly, rather than intervention driven specifically by the subarachnoid blood component in isolation.

Because this composite outcome combined cranial and vascular procedures, a sensitivity analysis was performed excluding the one endovascular case (*n* = 68; 11 events, 57 non-events). Results were materially unchanged: WBC AUC = 0.858 (95% CI 0.713–1.000), GCS AUC = 0.817 (95% CI 0.658–0.977), and the WBC + GCS combined model AUC = 0.898 (95% CI 0.772–1.000; sensitivity 90.9%, specificity 75.4%). In multivariable analysis, both WBC (OR = 4.005, 95% CI 1.492–10.747, *p* = 0.006) and GCS (OR = 0.380, 95% CI 0.194–0.745, *p* = 0.005) remained independently significant, closely matching the primary-cohort estimates. This indicates that the primary findings were not driven by inclusion of this single vascular-intervention case.

## 4. Discussion

In this retrospective cohort study of 69 adult ED patients with CT-confirmed tSAH, four parameters achieved statistically significant individual AUC values for predicting neurosurgical intervention: WBC, GCS, ISS, and neutrophil count. NLR and CRP did not demonstrate clinically meaningful discriminatory capacity in the acute triage setting. The primary combined model—WBC + GCS, both immediately available at first patient contact—achieved an AUC of 0.904, the highest combined diagnostic performance observed across all analyses conducted in this study. Pairwise DeLong testing confirmed this improvement did not reach statistical significance, a finding that likely reflects the limited statistical power of this small cohort rather than definitive evidence against an additive effect; nonetheless, the combined model’s incremental value over either predictor alone remains statistically unproven and should not be overstated. Importantly, in multivariable analysis both WBC (*p* = 0.006) and GCS (*p* = 0.002) were independently significant predictors, indicating that these two readily available parameters carry complementary and non-redundant prognostic information.

WBC emerged as the strongest individual predictor in this cohort (AUC = 0.850, cut-off > 11.20 × 10^3^/µL; sensitivity 91.7%, specificity 71.9%), closely followed by GCS and ISS. Because leukocytosis after major trauma can reflect a nonspecific physiological stress response correlated with overall injury burden, it is important to determine whether WBC provides information distinct from general trauma severity or merely acts as a surrogate for it. To address this, WBC and ISS—our two available indices of, respectively, inflammatory response and overall injury severity—were entered together in a multivariable model; WBC and ISS were only weakly correlated (r = 0.235), and both remained independently significant predictors of neurosurgical intervention when adjusted for one another (WBC OR = 3.949, 95% CI 1.595–9.779, *p* = 0.003; ISS OR = 2.610, 95% CI 1.238–5.502, *p* = 0.012). This finding suggests that WBC’s predictive value is not simply attributable to it being a proxy for overall injury severity, although we cannot fully exclude that WBC and ISS each capture only partially overlapping, non-specific components of physiological stress rather than a neuroinflammatory process specific to intracranial injury. The association between elevated admission WBC and neurosurgical intervention is biologically plausible and consistent with prior literature. Trauma induces a systemic inflammatory response characterized by rapid neutrophil mobilization and leukocytosis; the magnitude of this response broadly reflects injury severity and tissue destruction. Admission WBC has been reported as associated with the presence of significant intracranial lesions in blunt trauma [10]. In the context of subarachnoid hemorrhage, leukocyte accumulation within the first hours after injury has been associated with secondary complications, including a higher risk of in-hospital pneumonia, that may in turn worsen neurological outcome [11,12,19]. Our study-derived WBC cut-off of > 11.20 × 10^3^/µL aligns with the commonly used leukocytosis threshold and requires no additional cost or time beyond routine complete blood count.

The GCS represents the most widely utilized neurological assessment tool in clinical practice, particularly for the serial monitoring of TBI patients. Admission GCS was a strong individual predictor in the present cohort (AUC = 0.831, cut-off ≤ 14; sensitivity 83.3%, specificity 71.9%); notably, this univariable finding contrasts with at least one large registry study in which GCS was not an independent predictor of neurosurgical intervention after multivariable adjustment [18], suggesting that its predictive strength here may be attenuated once other factors are accounted for in larger, risk-adjusted cohorts. Age-adjusted recalibration of GCS has also been proposed to improve its performance for this specific outcome, as unadjusted GCS may under- or overestimate neurosurgical risk differently across age groups [20]. Dunham et al. reported that TBI patients undergoing surgical decompression had significantly lower admission GCS scores than non-operative patients, with the degree of GCS deficit more pronounced in patients not following commands at discharge [21]. GCS retained independent significance after adjustment for WBC in this cohort (OR = 0.331, 95% CI 0.167–0.656, *p* = 0.002), with a low inter-predictor correlation (r = −0.365) making multicollinearity an unlikely explanation. GCS and WBC appear to capture complementary dimensions of injury severity—neurological impairment and systemic inflammatory load, respectively—and their combination reflects this complementarity, supported by the independent significance of both components. It should be noted that pupillary reactivity was not incorporated in this analysis; the GCS-Pupils score (GCS-P), which subtracts a pupil reactivity score from the GCS, has been proposed as an extended index of clinical severity [22] and was recently validated as providing incremental prognostic value over GCS alone for mortality and unfavorable outcome in the large CENTER-TBI and TRACK-TBI cohorts [23]. Pupillary reactivity data were not systematically available in this retrospective dataset; whether GCS-P would outperform GCS in the specific context of predicting neurosurgical intervention in tSAH remains an open question for future prospective study.

ISS also performed well individually (AUC = 0.827, cut-off > 24; sensitivity 75.0%, specificity 87.7%). Notably, the study-derived ISS cut-off (>24) is considerably higher than the conventional threshold of >15 typically used to define major trauma, suggesting that in the specific context of tSAH, a substantially greater overall injury burden—beyond that required to meet general major-trauma criteria—is needed before the probability of neurosurgical intervention rises appreciably. This may reflect the fact that isolated tSAH with lower ISS values is more often accompanied by extracranial injuries of limited severity, whereas patients crossing the higher ISS threshold identified here are more likely to have severe concomitant polytrauma that itself increases the likelihood of associated neurosurgical pathology.

NLR (AUC = 0.572) and CRP (AUC = 0.581) did not achieve significant discriminatory capacity. A 2023 meta-analysis found that while NLR predicted adverse functional outcomes in TBI, it did not significantly predict surgical treatment [12]—consistent with our result. Notably, lymphocyte count itself was significantly higher, not lower, in the surgery group (2.55 vs. 1.70 × 10^3^/µL; *p* = 0.049; Table 1), alongside the expected elevation in neutrophil count (*p* = 0.001); this pattern is consistent with an acute stress-related leukocytosis affecting both leukocyte subpopulations in parallel, rather than the classic cortisol-mediated neutrophilia-with-lymphopenia pattern described in some critical illness literature. Because NLR is a ratio of two components that moved in the same direction in this cohort, much of each component’s discriminatory signal may have been attenuated in the ratio itself, which could explain why NLR was not significantly discriminatory despite both of its constituent counts differing significantly between groups. CRP, which typically begins to rise only around 6 h after the inciting stimulus and peaks at approximately 48 h, with a plasma half-life of about 19 h [24], is similarly too early to reflect the full inflammatory response at ED presentation. WBC and neutrophil count, driven primarily by rapid neutrophil mobilization, appear better suited for acute triage discrimination. It is also worth situating WBC within the broader landscape of emerging TBI biomarkers. A composite inflammatory-nutritional index, the neutrophil-to-albumin ratio (NAR), was recently reported to outperform NLR as a prognostic biomarker in TBI [25]; albumin data were not available in the present dataset, precluding calculation of NAR here. Separately, glial fibrillary acidic protein (GFAP) and ubiquitin C-terminal hydrolase-L1 (UCH-L1) are brain-specific biomarkers with high diagnostic accuracy for detecting intracranial hemorrhage after blunt head trauma [26]; unlike WBC and GCS, these require dedicated assay platforms not universally available at initial ED triage, so WBC and GCS remain a more immediately accessible, low-cost complement to these emerging, more specific but less widely deployed biomarkers, rather than a replacement for them.

Consistent with the literature, conservative management represents the predominant treatment approach in tSAH, and the rate of neurosurgical intervention observed in this study was correspondingly low. Diaz et al. reported that among 225 conscious tSAH patients and 826 non-SAH intracranial hemorrhage patients, the SAH cohort had a significantly lower neurosurgical intervention rate [27]. Similarly, Gates et al. reported that none of 67 tSAH patients required neurosurgical intervention in a tertiary referral cohort [28]. The 17.4% surgical rate observed in the present study is broadly consistent with the low overall intervention rates reported in the tSAH literature, although this comparison should be interpreted cautiously: reported rates vary substantially across cohorts—including a series in which no patients required intervention [28]—likely reflecting differences in injury severity distribution, referral patterns (tertiary versus community ED), and case-mix between populations, rather than a single universally expected rate. Even in conservatively managed patients, the natural history of tSAH may involve progressive hemorrhagic changes: Chieregato et al. reported that 46.8% of tSAH cases demonstrated some radiological progression on follow-up CT (24.1% showing significant progression by Marshall classification) [29], with hemorrhagic progression associated with unfavorable outcomes [29,30]. The pathophysiological basis for such progression may relate to the broader concept of early brain injury following subarachnoid hemorrhage, encompassing microvascular dysfunction and neuroinflammatory cascades that begin within the first hours after the bleed [31]. In contrast, a more recent, larger series (*n* = 340) restricted specifically to isolated tSAH reported a substantially lower radiographic progression rate of 5.6% [32], suggesting that progression rates may vary considerably depending on population selection (isolated versus all tSAH) and case mix, and that this remains an evolving area of investigation. This underscores the importance of early identification of high-risk patients—the central aim of the present study.

It is important to distinguish the present approach from established TBI prognostic models such as IMPACT and CRASH, and CT-based severity classifications such as the Marshall and Rotterdam scores. These validated tools were developed and externally tested primarily to predict mortality or long-term functional outcome (e.g., 6-month Glasgow Outcome Scale) in moderate-to-severe TBI, and, in the case of IMPACT and CRASH, they retain good discrimination but imperfect calibration—with a consistent tendency to overpredict mortality and unfavorable outcome—when re-evaluated in contemporary cohorts [33]; similarly, Marshall and Rotterdam CT-based classifications have shown variable prognostic utility for mortality prediction on systematic evaluation [34]. None of these established models was designed specifically to predict the acute, binary decision of whether neurosurgical intervention is required—the clinical question addressed in the present study. Only a small and recent body of literature has examined inflammatory hematological parameters specifically in relation to TBI patients undergoing decompressive craniectomy: Corbett et al. examined whether admission hematological abnormalities (including INR, fibrinogen, and neutrophil-to-lymphocyte ratio) offered additional prognostic value for 18-month neurological outcome beyond the IMPACT model in patients who had already undergone decompressive craniectomy for severe TBI, finding that this additional value was limited once IMPACT-predicted risk was accounted for [35], and a 2025 study found that incorporating NLR improved outcome-prediction accuracy in patients with acute traumatic subdural hematoma undergoing surgical treatment [36]. The present findings extend this limited literature to tSAH specifically, and suggest that WBC and GCS—both available before any CT-based severity score can be calculated—may offer a complementary, more immediately actionable alternative for the specific triage question of neurosurgical need, rather than a replacement for established outcome-prediction models. Detailed CT-based severity classifications such as the Marshall and Rotterdam scores, which some recent studies have combined with inflammatory markers to predict outcome, could not be calculated in the present retrospective dataset, as this would have required systematic re-review of original CT images for basal cistern status, midline shift, and lesion volume; ISS, as documented in the clinical record, was used as the available injury-severity measure instead. Beyond conventional CT-based scoring, emerging quantitative and functional imaging techniques for tSAH—including advanced MRI-based approaches capable of characterizing microstructural injury and perfusion disturbances—may eventually offer additional refinement in risk stratification, although their integration into acute clinical decision-making remains limited at present [37].

### Limitations

Several limitations must be acknowledged. First, the retrospective single-center design limits generalizability. Relatedly, the exact interval between injury and blood sampling was not individually recorded for each patient and could not be reported as a discrete variable. However, per institutional ED protocol, intravenous access and initial blood sampling are obtained immediately upon hemodynamic stabilization for all trauma patients; consequently, for hemodynamically stable patients—who constituted the large majority of this cohort—laboratory samples and initial CT imaging were both obtained within approximately the first hour of ED arrival, providing a reasonably narrow and consistent early time window across patients. Nonetheless, formal per-patient timestamps were not captured, and future prospective studies should explicitly record and standardize this interval. Second, the very small event count (*n* = 12) severely constrains multivariable modeling; the resulting EPV of 6.0 falls below the conventional threshold of 10 [38], and the correspondingly wide confidence intervals for both predictors (e.g., WBC 95% CI 1.473–10.597) indicate substantial estimation uncertainty despite nominal statistical significance; results should therefore be interpreted with caution and regarded as hypothesis-generating pending larger prospective validation. Third, all diagnostic cut-offs, including the ISS threshold of >24, were derived post hoc using the Youden index on the same dataset used for evaluation; external validation is required before clinical application. Fourth, ISS was available retrospectively rather than prospectively, limiting interpretation of its real-world triage utility. Fifth, patients younger than 18 years were excluded a priori to avoid physiological heterogeneity from differing pediatric reference ranges; this limits generalizability to pediatric tSAH populations, which may warrant separate investigation. Sixth, neutrophil and lymphocyte counts could not be evaluated in the primary multivariable model due to collinearity with WBC and the limited event count, and their independent contribution beyond WBC cannot be excluded. Seventh, radiological severity indices (e.g., subarachnoid blood volume, associated contusion or subdural/epidural hematoma, midline shift, basal cistern effacement) and clinical factors such as anticoagulant/antiplatelet use and serial neurological examination findings were not available; these are recognized determinants of the decision to intervene surgically, and their omission raises the possibility of residual confounding, such that the independent associations reported here should not be interpreted as causal. Eighth, model calibration (e.g., calibration plot, Brier score) was not assessed, as the very low event count would have made such estimates unstable and difficult to interpret meaningfully. Bootstrap internal validation was performed and indicated modest optimism (Section 3.3); nonetheless, the reported AUC and ORs reflect discrimination in a single retrospective cohort, and no external validation has been performed. Ninth, GFAP and UCH-L1—brain-specific biomarkers increasingly used in TBI triage—could not be evaluated in this cohort, as they were not measured in this retrospective dataset; their comparative performance against WBC and GCS for the specific endpoint of neurosurgical intervention remains an open question for future prospective study. Tenth, as detailed in Section 3.5, the great majority of neurosurgical interventions in this cohort were performed in the presence of a structural lesion accompanying the tSAH (e.g., hematoma, subdural hemorrhage, or mass effect) rather than for isolated subarachnoid blood; one patient additionally had a demonstrated cerebral aneurysm, raising the possibility of an aneurysmal contribution to the hemorrhage that cannot be fully excluded on retrospective imaging review. Consequently, the present models should be understood as predicting the need for neurosurgical intervention in tSAH-inclusive TBI presentations as a whole—reflecting the reality that isolated tSAH rarely constitutes the sole indication for surgery—rather than intervention attributable to the subarachnoid component specifically; this distinction should be explicitly considered before any attempt to generalize these findings to a hypothetical population with radiologically isolated tSAH.

## 5. Conclusions

In this single-center exploratory cohort of 69 adult tSAH patients, WBC, GCS, ISS, and neutrophil count were significantly associated with neurosurgical intervention. The WBC + GCS combined model showed numerically higher discrimination (AUC = 0.904) than either predictor alone, and both WBC and GCS remained independently associated with the outcome in multivariable analysis; however, the incremental AUC difference over each individual predictor was not statistically significant by DeLong testing, and this finding should be interpreted as hypothesis-generating rather than as demonstrated evidence of additive predictive value. NLR and CRP did not demonstrate useful acute discriminatory capacity. Given the very small event count and the absence of internal or external validation, the reported AUC and odds ratios represent apparent, in-sample performance rather than a validated estimate of real-world diagnostic accuracy. A WBC threshold exceeding 11.20 × 10^3^/µL, combined with GCS assessment, may serve as a candidate tool for early stratification of adult tSAH patients, pending validation in larger, ideally multicenter, prospective cohorts.

## Figures and Tables

**Table 1:** Baseline characteristics by neurosurgical intervention status. Values are median (IQR) or *n* (%). *p*-values from the Mann–Whitney U test (continuous) or Fisher’s exact test (categorical).

Variable	Total (*n* = 69)	Surgery (*n* = 12)	No Surgery (*n* = 57)	*p*
Demographic				
Age, years	65.0 (42.0–78.0)	55.0 (42.0–69.5)	68.0 (43.0–79.0)	0.289
Male, *n* (%)	45 (65.2)	9 (75.0)	36 (63.2)	0.521
Diabetes mellitus, *n* (%)	17 (24.6)	2 (16.7)	15 (26.3)	0.716
Hypertension, *n* (%)	34 (49.3)	7 (58.3)	27 (47.4)	0.540
Active cancer, *n* (%)	1 (1.4)	0 (0.0)	1 (1.8)	1.000
Trauma mechanism				
Same-level fall, *n* (%)	39 (56.5)	4 (33.3)	35 (61.4)	0.265 ^a^
Fall from height, *n* (%)	13 (18.8)	4 (33.3)	9 (15.8)	
Pedestrian traffic accident, *n* (%)	7 (10.1)	2 (16.7)	5 (8.8)	
Assault, *n* (%)	8 (11.6)	1 (8.3)	7 (12.3)	
Other, ^b^ *n* (%)	2 (2.9)	1 (8.3)	1 (1.8)	
Laboratory parameters				
Neutrophil, 10^3^/µL	6.00 (4.56–10.67)	12.55 (7.36–16.13)	5.67 (4.19–8.89)	0.001
Lymphocyte, 10^3^/µL	1.80 (1.30–2.70)	2.55 (1.50–4.45)	1.70 (1.30–2.50)	0.049
NLR	3.33 (1.91–5.82)	4.33 (1.88–9.69)	3.33 (1.99–5.00)	0.443
WBC, 10^3^/µL	9.40 (7.50–13.60)	15.15 (12.60–18.02)	8.60 (7.40–11.40)	<0.001
CRP, mg/L	2.31 (1.00–7.72)	6.50 (0.90–17.70)	2.31 (1.00–6.20)	0.384
Clinical scores				
Admission GCS	15.0 (13.0–15.0)	7.0 (3.0–14.0)	15.0 (14.0–15.0)	<0.001
ISS	17.0 (11.0–22.0)	28.0 (22.8–36.3)	16.0 (11.0–19.0)	<0.001

^a^ Overall *p*-value for trauma mechanism distribution across groups. ^b^ Other: head trauma after seizure (*n* = 1), scooter accident (*n* = 1). IQR: interquartile range; NLR: neutrophil-to-lymphocyte ratio; WBC: white blood cell count; CRP: C-reactive protein; GCS: Glasgow Coma Scale; ISS: Injury Severity Score.

**Table 2:** ROC analysis: diagnostic performance and pairwise AUC comparisons. AUC with 95% CI (Hanley–McNeil method). Cut-offs by Youden index (study-derived, exploratory). ^a^ DeLong test vs. WBC + GCS combined model.

Variable	AUC	95% CI	Cut-Off	Sens.	Spec.	*p* ^a^
WBC, 10^3^/µL	0.850 *	0.708–0.992	>11.20	91.7%	71.9%	0.571
Admission GCS	0.831 *	0.683–0.980	≤14	83.3%	71.9%	0.455
ISS	0.827 *	0.678–0.977	>24	75.0%	87.7%	—
Neutrophil, 10^3^/µL	0.807 *	0.651–0.963	>7.10	83.3%	68.4%	—
CRP, mg/L	0.581	0.397–0.765	>11.01	50.0%	84.2%	—
NLR	0.572	0.387–0.756	>5.82	50.0%	78.9%	—
WBC + GCS (primary)	0.904 *	0.785–1.000	—	75.0%	93.0%	Ref.
WBC + ISS (secondary)	0.886 *	0.759–1.000	—	100.0%	64.9%	—

* AUC significantly > 0.5 (*p* < 0.05). ISS and GCS were not combined due to high collinearity (r = −0.680). WBC + GCS (r = −0.365) and WBC + ISS (r = 0.235) both had acceptable collinearity.

**Table 3:** Logistic regression analysis. OR per 1 SD; 95% CI by Wald test (Fisher information matrix). EPV: events per variable.

Predictor	OR	95% CI	*p*
Univariable analysis			
WBC, 10^3^/µL	3.907	1.696–9.000	0.001
GCS	0.302	0.160–0.567	<0.001
Neutrophil, 10^3^/µL	3.216	1.599–6.469	0.001
ISS	2.735	1.381–5.418	0.004
Lymphocyte, 10^3^/µL	1.787	1.023–3.125	0.042
CRP, mg/L	1.382	0.844–2.263	0.198
NLR	1.359	0.777–2.377	0.282
Age, years	0.751	0.407–1.387	0.361
Multivariable—primary model (WBC + GCS, EPV = 6.0)			
WBC, 10^3^/µL	3.951	1.473–10.597	0.006
Admission GCS	0.331	0.167–0.656	0.002

Combined model AUC = 0.904 (95% CI 0.785–1.000); sensitivity 75.0%, specificity 93.0%. Secondary model (WBC + ISS): WBC OR = 3.949 (95% CI 1.595–9.779, *p* = 0.003); ISS OR = 2.610 (95% CI 1.190–5.502, *p* = 0.012).

**Table 4:** Neurosurgical intervention procedures and indications (*n* = 12).

Intervention Category	*n* (%)	Procedure(s)	Primary Indication (Per CT/Operative Report Review)
Isolated cranial neurosurgical procedure	8 (66.7%)	Craniotomy, craniectomy, EVD placement, hematoma evacuation, or aneurysm clipping	Structural lesion accompanying tSAH (intraparenchymal/intraventricular hematoma, subdural hematoma, or significant mass effect)
Cranial neurosurgical procedure + concurrent orthopedic procedure	3 (25.0%)	Cranial procedure as above, combined with orthopedic surgery for a separate extracranial injury	Structural lesion accompanying tSAH, as above; orthopedic component addressed a co-existing fracture
Therapeutic intracranial vascular intervention (no craniotomy/craniectomy/EVD)	1 (8.3%)	Carotid digital subtraction angiography with endovascular treatment	Trauma-related vascular pathology directly associated with the tSAH

EVD: external ventricular drain; tSAH: traumatic subarachnoid hemorrhage. One patient in the isolated-cranial-procedure category (File 1622235) additionally had a cerebral aneurysm identified on angiography (see Discussion, Limitations point 10).

## Data Availability

The data presented in this study are available on request from the corresponding author, subject to institutional and ethical restrictions on patient data sharing.

## Supplementary Materials

The following supporting information can be downloaded at: https://www.mdpi.com/article/10.3390/diagnostics16182949/s1, Figure S1: Patient-selection flow diagram. Of 1884 patients with neurosurgical consultation requested in the Emergency Department during the study period, 148 had CT-confirmed traumatic subarachnoid hemorrhage (tSAH); 71 were excluded for incomplete medical records, active infection/autoimmune disease, or corticosteroid/immunosuppressant use, and a further 8 pediatric patients were excluded, yielding the final analytic cohort of 69 adult patients (12 surgery, 57 no-surgery).
