# Supplementary material for: Does the Integration of Inflammatory Markers with Clinical Scoring Systems Improve the Prediction of Neurosurgical Intervention in Emergency Department Patients Diagnosed with Traumatic Subarachnoid Hemorrhage?
Source: Diagnostics (Basel). 2026 Sep 12;16(18):2949. doi: 10.3390/diagnostics16182949 (PMC13605286; doi:10.3390/diagnostics16182949)

Supplementary Material

Supplementary Figure S1. *Patient-selection flow diagram. Of 1,884 patients with neurosurgical consultation requested in the Emergency Department during the study period, 148 had CT-confirmed traumatic subarachnoid hemorrhage (tSAH); 71 were excluded for incomplete medical records, active infection/autoimmune disease, or corticosteroid/immunosuppressant use, and a further 8 pediatric patients were excluded, yielding the final analytic cohort of 69 adult patients (12 surgery, 57 no-surgery).*

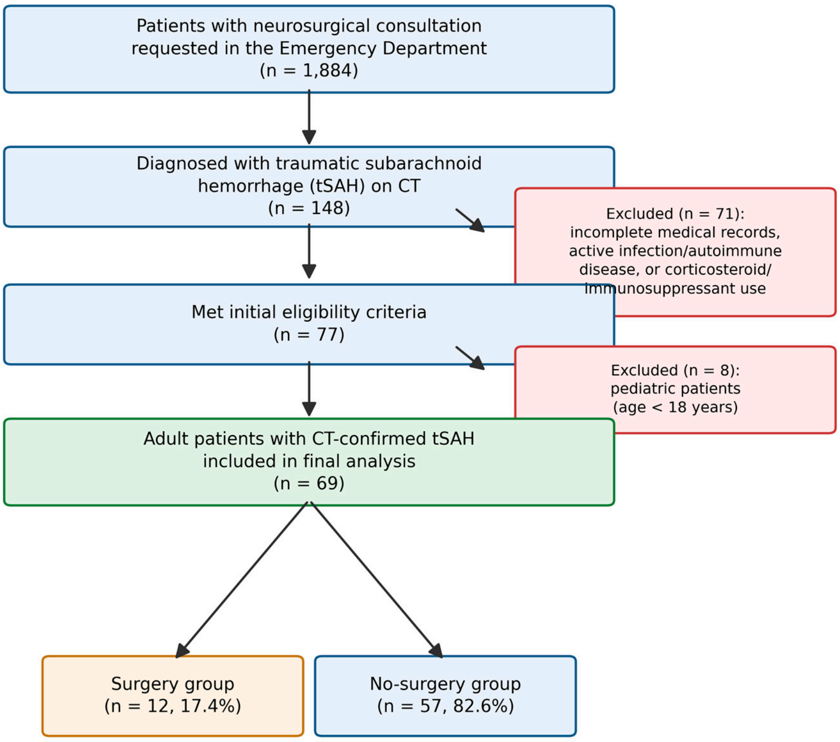

Supplement: Supplementary file 1 [file diagnostics-16-02949-s001.zip › diagnostics-4542676-supplementary.pdf]
